# An improved solid medium-based culturing method for *Steinernema hermaphroditum*

**DOI:** 10.17912/micropub.biology.001110

**Published:** 2024-01-27

**Authors:** Nathan Y. Rodak, Chieh-Hsiang Tan, Paul W. Sternberg

**Affiliations:** 1 Division of Biology and Biological Engineering, California Institute of Technology, 1200 East California Boulevard, Pasadena, CA 91125, USA; 2 Current Address: Case Western Reserve University, Cleveland, OH 44106, USA

## Abstract

*Steinernema hermaphroditum* is the only identified entomopathogenic nematode that is consistently hermaphroditic and thus offers a great opportunity to use genetic approaches to probe symbiosis. Evolutionarily, ecologically, and morphologically distinct from laboratory nematodes commonly used in the laboratory, with both forward and reverse genetics tools available, this species also provides an opportunity to explore other areas of biology, especially using comparative studies. Here, we describe an improved solid medium-based culturing method for *S. hermaphroditum* that we found particularly helpful for phenotypic analysis and genetic manipulation. We document the rapid increase in the size of the worm; and show that the uniform growth of the worm on this medium provides a good basis for developmental studies. Finally, we measure the brood size of individual animals, which, although far larger, has a very similar trajectory to that of the hermaphroditic *Caenorhabditis elegans,* suggesting common reproductive restraints.

**Figure 1. An improved solid medium culturing method for Steinernema hermaphroditum.**
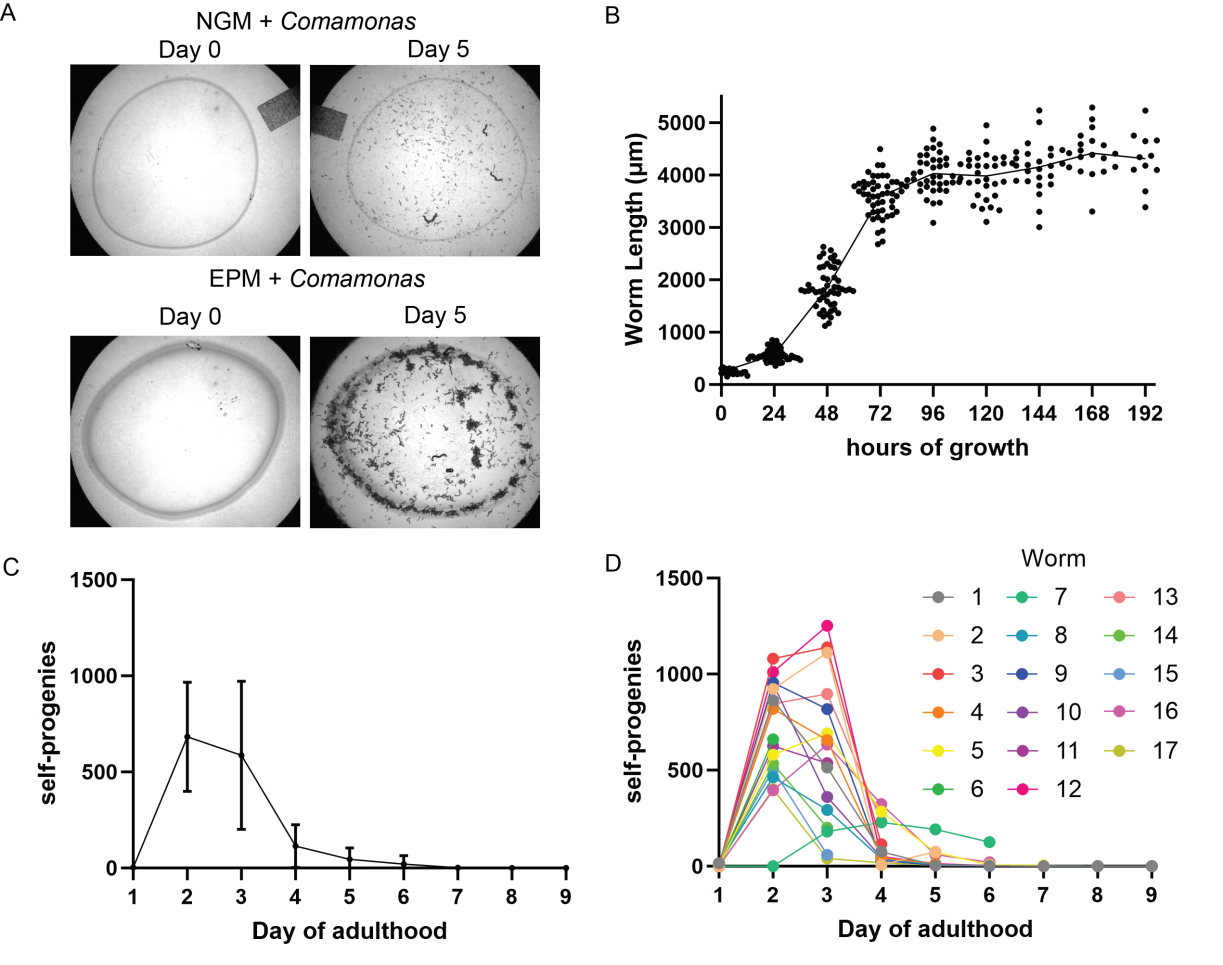
(A) Enriched Peptone Medium (EPM) agar plates seeded with *Comamonas aquatica* can sustain a much larger population of *S. hermaphroditum* than NGM agar. Starting with three young larvae, the resulting population exhausted a lawn of *C. aquatica* bacteria grown on NGM by day 5 (the mark left by the lawn edge can still be seen), while the *C. aquatica* lawn grown on EPM agar was still present and was supporting F _1_ animals that had grown much larger in size. Pictures are representative of six replicates. (B) *S. hermaphroditum* grew rapidly but uniformly on *C. aquatica* /EPM plates. Starting from a near-synchronous population, with an average length of 241 ± 48 µm (n = 42), the worms developed rapidly, increasing in length to 571 ± 119 µm (n = 52) at 24 hours, 1844 ± 384 µm (n = 52) at 48 hours, 3592 ± 373 µm (n = 51) at 72 hours, 4028 ± 371 µm (n = 39) at 96 hours, 3982 ± 423 µm (n = 31) at 120 hours, 4152 ± 472 µm (n = 25) at 144 hours, 4416 ± 437 µm (n = 19) at 168 hours, and 4312 ± 512 µm (n = 11) at 192 hours. (C) *S. hermaphroditum* hermaphrodites produced 1363 ± 623 self-progeny (n = 17) with 3 ± 6 (n = 17) on day 1, 683 ± 284 (n = 17) on day 2, 587 ± 385 (n = 16) on day 3, 115 ± 111 (n = 13) on day 4, 45 ± 59 (n = 10) on day 5, 21 ± 43 (n = 8) on day 6, 2 ± 2 (n = 5) on day 7, 0 (n = 4) on day 8, 0 ± 1 (n = 4) on day 9. Values are mean ± SD (17 worms were analyzed; for each time point, the data from all the surviving worms were used). (D) Brood size analysis of individual animals. Each color point represents the number of self-progeny produced by an individual animal during the day of adulthood indicated on the X axis, with identically colored lines connecting the colored dots. Data from the same animals are presented as summaries in in C and as individuals in D. All worms were incubated at 25°C.

## Description

The nematode *Steinernema hermaphroditum* (Griffin *et al.* 2001; Stock *et al.* 2004; Bhat *et al.* 2019), is the only entomopathogenic nematode characterized that is consistently hermaphroditic (Cao *et al.* 2022), a reproductive mode that has been critical for the successful use of most of the common laboratory cultured nematodes in molecular genetics research (Felix 2006; Sommer 2006; Gupta *et al.* 2007; Corsi *et al.* 2015). A genetically tractable entomopathogenic nematode is a highly attractive animal model for symbiosis research, as they are not only parasitic to insect hosts but also themselves the host in a mutualistically symbiotic relationship with their pathogenic bacteria that help them kill their insect prey. In addition to its relationships with its insect host and its bacterial endosymbiont, the biology of *S. hermaphroditum* offers other opportunities, particularly for developmental studies. Specifically, as a clade IV nematode, it is evolutionarily distant from most of the small free-living nematodes commonly used as genetic models (such as the clade V *Caenorhabditis elegans* and *Pristionchus pacificus* ), and it is morphologically distinctively different from these nematodes. Our laboratory has shown that *S. hermaphroditum* is genetically tractable, with tools developed for both forward and reverse genetic analyses (Cao *et al.* 2022; Cao 2023; Schwartz *et al.* 2023), and efforts have been made to adapt or establish experimental methods with *S. hermaphroditum* as the animal model (Garg *et al.* 2022; Alani *et al.* 2023; Huynh *et al.* 2023).

Previously, we showed that *S. hermaphroditum* could be cultured on bacterial lawns grown on Nematode Growth Medium (NGM) agar in Petri plates (Cao *et al.* 2022), using as the bacteria either its native symbiont *Xenorhabdus griffiniae* HGB2511 (Cao et al. 2022) or *Comamonas aquatica* DA1877 (Avery and Shtonda 2003; Watson *et al.* 2014), on which the worm grows faster. Culturing *S. hermaphroditum* on *C. aquatica* offers several advantages. First, unlike *Xenorhabdus* , *C. aquatica* is not pigmented, offering an unobstructed view of the transparent *S. hermaphroditum* large and small, resembling the experience of examining *C. elegans* growing on *Escherichia coli* OP50 and is particularly helpful for developmental and phenotypic analysis. Second, *C. aquatica* may provide a less challenging environment for *S. hermaphroditum* than does *X. griffiniae* ; for example, worms recover better on *C. aquatica* after microinjection (Cao 2023; Schwartz *et al.* 2023). Third, *Xenorhabdus* bacteria are known to exist in two forms (Akhurst 1980) and are light sensitive (Xu and Hurlbert 1990) ; both characteristics may cause unintended variations in experiments if not well controlled.

However, we found that NGM is a poor medium for *C. aquatica* , and the poor growth led to inefficient food for effective maintenance of the worm, exacerbated by the body size and the brood size of the animal (see below). In addition, we observed that worms cultured on *C. aquatica* /NGM frequently wandered off the bacterial lawn and left the plate entirely, likely also a result of poor bacterial growth conditions. We thus experimented with other media, resulting in conditions that significantly abrogated this problem (see below).

We adopted the Enriched Peptone Plates with nystatin recipe from Evans (2006) with agar concentration modified to 1.8% (weight/volume) (See Methods for the complete protocol), which we abbreviate as EPM (Enriched Peptone Medium). EPM supports a much higher level of *C. aquatica* growth than NGM, but the bacterial lawn remained highly transparent and was evenly grown. *C. aquatica* /EPM overcomes the limitations of *C. aquatica* /NGM, as it supports a far larger number of animals per plate, and the vast majority of the animals stay on the lawn. As in the example shown in Fig. 1A, in which 3 larvae were placed on either a 10 cm NGM plate or a 10 cm EPM plate seeded with *C. aquatica* , the EPM plates allowed the F _1_ animals to develop to a larger size in comparison to the NGM plates, on which the food was more quickly exhausted, making EPM plates more useful for genetic analysis. If cultured on DA1877 on EPM at 25°C, the size of *S. hermaphroditum* hermaphrodites increased rapidly, reaching an average length of about 4 mm, with some individuals exceeding 5 mm ( Fig. 1B ). Animal growth over time showed little variation ( Fig. 1B ), providing a good basis for developmental studies. The lack of bacterial pigmentation and ample food supply facilitated quantification of the self-fertilizing brood size of *S. hermaphroditum* . On average, an unmated hermaphrodite has 1,363 ± 623 progeny (n = 17) ( Fig. 1C ), with some animals having a brood size exceeding 2,000 ( Fig. 1D ). Compared to the rather uniform size increase, larger variations were observed between individual animals. Nonetheless, it is clear that peak productivity and the vast majority of reproduction occurs on the second and third day of adulthood and drops precipitately afterwards. This resembles the time course of selfing reproduction seen in the clade V hermaphroditic nematode *C. elegans* (Maupas 1900; Hirsh *et al.* 1976; Gems and Riddle 1996; Hughes *et al.* 2007), which may indicate a shared reproductive constraint. As with most hermaphroditic nematodes described (Maupas 1900; Felix and Sternberg 1996; Sommer *et al.* 1996; Pires-daSilva 2007; Shinya *et al.* 2014), *S. hermaphroditum* hermaphrodites have a somatic body similar morphologically to the females of related species, suggesting they have a female origin. The brood size of *C. elegans* by self-fertilizing was shown very early to be restricted by sperm (Maupas 1900) , which are only produced during late larval development, after which the germline shifts from spermatogenesis to oogenesis (Hirsh *et al.* 1976; Ward and Carrel 1979). The sperm followed by oocyte pattern was also followed by the hermaphrodite of other androdioecious (hermaphrodite with, often rare, males) species (Rudel *et al.* 2005; Shinya *et al.* 2014), but alternative patterns are possible, as has been shown in the hermaphrodite of trioecious *Auanema* species, of which sperm are produced continuously (McCaig *et al.* 2017). The similar trend in progeny production we observed in *S. hermaphroditum* led us to reason that hermaphroditism in this species also involves the production of sperm in the gonad before shifting to the production of oocytes, and a major restriction on the brood size was the number of sperm available. We also observed large numbers of unfertilized oocytes laid by post-reproductive hermaphrodites, as has previously been seen in post‑reproductive *C. elegans* (Maupas 1900; Hirsh *et al.* 1976; Ward and Carrel 1979), further suggesting that the hermaphroditic reproduction of this clade IV nematode is similar to that of the clade V nematode *C. elegans* , despite their phylogenetic distance and difference in brood size.

In summary, we have developed improved culturing methods for *S. hermaphroditum* that we believe could facilitate the adaptation of the animal as a model, particularly for laboratories already familiar with techniques for working with *C. elegans.* We analyzed the growth and reproduction of *S. hermaphroditum* using this culture method, both to provide a baseline for the method and because using previously existing methods we had found some analyses to be more difficult than they should be. In doing so, we showed that the brood size of unmated *S. hermaphroditum* hermaphrodites is very large (reaching more than 2,000 in some worms), is still quite variable (with some variation likely resulting from the early death of some worms from eggs hatching internally; “bagging”) and is likely limited by the number of sperm the hermaphrodite produces. Most importantly, we hope we have shown that *S. hermaphroditum* is a very different nematode but can be easily adopted by any lab with experience working with *C. elegans* .

## Methods

**Solid medium-based culturing method for *S. hermaphroditum***

Our recipe for Enriched Peptone Medium (EPM) agar was adapted from Evans (2006) with a minor modification (decreased agar concentration) and briefly described in Schwartz *et al.* (2023), in which the Enriched Peptone Medium EPM was utilized for culturing worms for microinjections and recovery afterwards. Briefly, for 1 liter:

(1) Weigh 1.2 g of sodium chloride (NaCl), 20 g of peptone (Bacto Peptone, Gibco), 18 g of agar (Bacto Agar, BD Diagnostics), and add deionized water to 1 liter.

(2) Autoclave. Let cool down to 55°C with stirring.

(3) Add solutions sterilized by autoclaving or filtration: 1 ml cholesterol (5 mg/ml in ethanol), 1 ml 1 M magnesium sulfate (MgSO _4_ ), 25 ml 1 M KPO _4_ (pH 6.0), and 10 ml of Nystatin suspension (10,000 unit/mL, N1638, Sigma)

For maintenance of *S. hermaphroditum* , 10 cm Petri plates are recommended.

Nematode Growth Medium (NGM) was prepared similarly to what was described by Brenner (1974). *Comamonas aquatica* DA1877 (Avery and Shtonda 2003; Watson *et al.* 2014) were cultured overnight in Luria-Bertani (LB) medium in an Erlenmeyer flask at 200 rpm, 37°C, and seeded on EPM or NGM agar in Petri plates the next day. Except when otherwise noted, all *S. hermaphroditum* were cultured at 25°C on Petri plates containing EPM agar with a lawn of *C. aquatica* DA1877.

**Obtaining a synchronized population of *S. hermaphroditum***

To obtain a near-synchronous population of young *S. hermaphroditum* , we modified the synchronization method based on Stiernagle (2006) and what we described in Cao *et al.* (2022). Briefly, gravid adults were washed off from the plates using M9 buffer (For 1 liter: 3 g KH _2_ PO _4_ , 6 g Na _2_ HPO _4_ , 5 g NaCl, and with 1 ml 1 M MgSO _4_ added after autoclaving) and collected into a 15 ml centrifuge tube. The worm-suspension was then centrifuged to remove the supernatant and water was added to the worm pellet to a total volume of 3.5 ml. 0.5 ml of 5 M NaOH and 1 ml of household bleach (8% available chlorine) were then added to the solution. The solution was mixed by gently shaking and allowed to react for about 5 minutes; most of the tissues besides the embryos should be dissolved by this point; longer reactions substantially decreased the survivability of the embryos. After this, the embryos were collected by centrifugation and washed with 10 ml of M9 buffer three times. After the washes, the embryos were seeded on NGM agar plates without bacteria and allowed to hatch overnight, and the larvae were collected the next day for experimentation.

**Comparison of *S. hermaphroditum* culture on *C. aquatica* /NGM and *C. aquatica* /EPM**

Growth comparisons between the media, as shown in Fig. 1A, were done by placing three semi -synchronized larvae on each of six 10 cm Petri plates containing *C. aquatica* /NGM and six 10 cm Petri plates containing *C. aquatica* /EPM ( Fig. 1A, left ). The plates were then assayed every 24 hours, and images were taken at the start of the experiment and on the fifth day after the start of the experiment, at which point the *C. aquatica* lawns on all six NGM plates were exhausted but were still present on all six EPM plates ( Fig. 1A, left ). Similar results were also observed in another independent trial.

**Growth rate analysis**

To measure the growth of *S. hermaphroditum***** hermaphrodites on *C. aquatica* /EPM plates, five semi-synchronized larvae each were transferred onto *C. aquatica* /EPM plates, and images were acquired every 24 hours using WormLab (MBF Bioscience, Williston, VT) equipment and software. The camera was a Nikon AF Micro 60/2.8D with zoom magnification. Animals were moved to new plates upon the hatching of progeny, and plates that included males were excluded from further analysis. Worm length was measured by processing the image with ImageJ (Fiji) (Schindelin *et al.* 2012) by converting the image to 8-bit, thresholding to obtain the shape of the worm, and by using the “skeletonize” and “analyze skeleton” feature (Arganda‐Carreras *et al.* 2010). Most worms, but not every worm, were measured at each time point.

**Brood size analysis**

J4 worms were placed individually on *C. aquatica* /EPM plates and maintained at 25°C. Every 24 hours, mothers were examined and transferred to fresh plates to remove them from the eggs they had laid. One day after the mother had been removed the progeny they had deposited were counted, removing each animal as it was counted using a Pasteur pipette connected to a vacuum aspirator to avoid double counting; plates were then checked again on the next day for overlooked progeny.
